# A high density of PD-L1-expressing immune cells is significantly correlated with favorable disease free survival in nonmetastatic colorectal cancer

**DOI:** 10.1097/MD.0000000000028573

**Published:** 2022-01-21

**Authors:** Ya-Ting Kuo, Chun-Kai Liao, Tse-ching Chen, Chen-Chou Lai, Sum-Fu Chiang, Jy-Ming Chiang

**Affiliations:** a Division of Colon and Rectal Surgery, Department of Surgery, Chang Gung Memorial Hospital, Lin-Kou Medical Center, Taoyuan, Taiwan; b Chang Gung University, College of Medicine, Taoyuan, Taiwan; c Department of Pathology, Chang Gung Memorial Hospital, Lin-Kou Medical Center, Taoyuan, Taiwan.

**Keywords:** CD8, colorectal cancer, PD-1, PD-L1, prognosis, tumor-infiltrating lymphocyte

## Abstract

The impact of immune cells (ICs) expressing various markers remains poorly understood in nonmetastatic colorectal cancer patients who have undergone colectomy. Here, we aimed to clarify the correlation between IC density and clinical parameters and survival.

Programmed death protein-1 (PD-1), programmed cell death protein ligand-1 (PD-L1), clusters of differentiation (CD)-3, CD-8, and CD45RO immunostaining was performed for 421 patients using tissue microarray and automatic counting. Tumor stroma area immune density was assessed in comparison to clinical histological factors and surgical outcomes.

High-density CD-8 expression was significantly associated with current smoking habits or a smoking history (*P* = .006). High-density of PD-1 expression was correlated with Lynch syndrome patients (*P* < .001) and with patients who did not consume alcohol (*P* = .034). A significant decrease in CR45RO expression density was associated with aging (*P* = .002 and *r* = –0.014), and high-density CD-3, CD-8, and PD-1 expression was significantly associated with right colon tumor location (*P* < .001). High CD-3 and PD-L1 expression was significantly associated with early tumor T-staging (*P* = .018 and *P* = .002). High-density PD-1 expression was significantly correlated with mucinous type adenocarcinoma (*P* = .027) and poor differentiation (*P* < .001). For treatment outcomes, multivariate analysis confirmed that patients exhibiting high-density PD-L1 expression possessed significantly longer disease free survival (adjusted hazard ratio: 0.752, 95% confidence interval [CI]: 0.61–0.92, *P* = .006) and overall survival (adjusted hazard ratio: 0.872, 95% CI: 0.75–1.91, *P* = .064)

Significantly varied density in IC subsets was related to distinct demographic or clinic-histological factors. The presence of high-density PD-L1-expressing ICs is an independent favorable prognostic factor for disease free survival and overall survival among stage I to III colorectal cancer patients.

## 1 Introduction

Recently, it has been established that differences in tumor microenvironment cells can be used to predict survival in patients diagnosed with colorectal cancers (CRCs).^[1–4]^ Tumor-infiltrating lymphocytes (TILs) together with stromal cells can function as an activated immune system against the cancer. TILs have been reported to be indicative of anticancer immunity and to correlate with the treatment outcome and survival in patients with CRC.^[1–3]^ However, different subsets of TILs are associated with various specific functions, and a variety of these subsets can improve therapeutic outcomes for CRC patients.^[4–8]^

In contrast, immune escape by cancer cells has been increasingly reported in recent years. Continuous exposure to tumor antigens results in the exhaustion of immune cells (ICs) and leads to poor clinical outcomes.^[2]^ The programmed cell death-1 (PD-1) and programmed cell death protein ligand-1 (PD-L1) pathways represent major mechanisms controlling immune suppression within the tumor microenvironment.^[9]^ PD-L1+ tumor cells can deliver an inhibitory signal to PD-1+ T lymphocytes that results in immune suppression.^[9]^ However, PD-L1 is not only expressed in CRC tumor cell but is also expressed in lymphocyte cells where it could exert a different impact.^[7,10–12]^ Based on this, the complex interrelationship between prognosis and PD-1/PD-L1 expression by ICs remains unclear.

Additionally, the majority of previous studies focused on the prognostic roles of ICs in regard to metastatic CRC.^[4–8,10–12]^ The impact of these cells on nonmetastatic (stage I–III) CRC in patients who underwent curative colectomy is limited. In this retrospective study, we therefore aimed to explore the demographic (age, sex, alcohol consumption, and smoking), clinic-pathologic factors, and treatment outcomes in relation to the different subsets of ICs found in stage I to III CRC patients by using tissue microarrays (TMAs), computer-assisted imaging, and automatic counting to analyze the densities of immunostained clusters of differentiation (CD)3+, CD8+, CDRO45+, PD-1+, and PD-L1+ markers in intra-tumor ICs.

## 2 Material and methods

Formalin-fixed paraffin-embedded (FFPE) histological sections of surgically resected primary colorectal tumors were obtained from 421 patients who underwent surgery with curative intent from 2012 to 2014 at the Chang Gung Memorial Hospital. Tumor staging was performed according to the staging system from the 7th American Joint Committee on Cancer. This study was approved by the Chang Gung Memorial Hospital Institution Review Board (CGMH IRB103-3326B/105-6652D).

### 2.1 Immunohistochemistry

TMA were performed using formalin-fixed paraffin-embedded specimens. TMA sections were prepared from each tumor. TMAs were constructed from representative tumor blocks for each case using an automated arrayer. In detail, four 1.0-mm tissue cores obtained from the center and periphery (core diameter 1 mm) were carefully sampled from each tumor paraffin block to appropriately represent potential tumor heterogeneity.

For immunohistochemical analyses, TMA slides were steam-heated for 30 minutes in pH 6 citrate buffers, and subsequent immunostaining was performed using a 25 min incubation period with the primary antibody (DakoAutostainer, Denmark). After optimizing the immunohistochemical conditions, the slides were deparaffinized, and immumo-histochemitry (IHC) was performed using an automated staining system (BOND-MAX; Leica Microsystems). The specimens were stained using antibodies specific for PD-1 (EH12.1 from BD Biosciences/Pharmingen; catalog no. 561273 or Abcam, ab 52587, mouse IgG1, clone NAT 105, 1:50), PD-L1 (Cell Signaling Technology, #13684 clone E1L3N, rabbit IgG1, 1:500, SP142, Spring Biosciences; catalog no. M4420; SP263, Roche/Ventana Medical Systems; catalog no. 790-4905), CD-3 (polyclonal rabbit anti-human; Dako North America Inc.; catalog no. A0452), CD-8 (monoclonal mouse anti-human; Dako North America Inc.; catalog no. M7103), and CD45RO (Dako, M7240, clone MIB-1, mouse IgG1, 1:100). Immunoreactions were visualized through the use of a biotinylated secondary antibody (LSAB/AP, #K5005 Dako). Finally, TMAs were counterstained with hematoxylin and covered with Cytoseal (Thermo Scientific, USA). All slides were then stained with hematoxylin and eosin (H&E). Slides stained for CD3, CD8, CD45RO, PD-1, and PD-L1 cells were first evaluated by a clinical pathologist. For antibody concentration, the dilution factor of the antibodies was optimized by using normal colon mucosa as negative and a positive control as recommended.

### 2.2 Image capture and automated quantification

**Figure 1:**
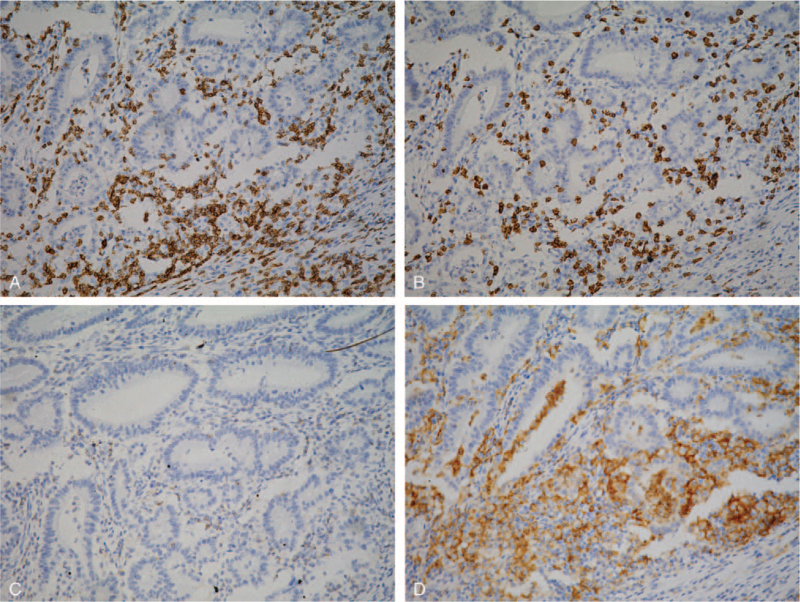
Representative examples of immunostaining of various markers of positive (brown) immune cells in CRC tissue microarrays. CD3 (A), CD8 (B), PD-1 (C), and PD-L1 (D) immune cells are shown. Negative immune cells are indicated by blue immunostaining. Digital images were analyzed using the image software, and the densities of positive immune cells (CD3, CD8, CD45RO, PD-1, and PD-L1 cells) were recorded as the number of positive cells per 1 mm^2^ of tissue surface area. CRC = colorectal cancer, PD-1 = programmed death protein-1, PD-L1 = programmed cell death protein ligand-1.

To measure the IHC expression of the different markers and to quantify the ICs, automated in situ enumeration of T lymphocytes within the colorectal mucosa was performed. We used the IHC membrane algorithm from Leica Technologies, Inc. (Vista, CA) to quantify membrane staining to determine the number of different marker-positive immune cells/mm^3^. For membrane staining, various input parameters were optimized and validated for each tissue sample and for the isotype controls. The automated-derived counts were used to calculate cells/mm^3^. Briefly, the slides containing immunohistochemically stained TMA sections were digitally scanned at a 200× magnification using a ScanScope Aperio AT Turbo slide scanner (Leica Microsystems). The images were visualized using the ImageScope software program (Leica Microsystems) and analyzed using the Aperio Image Toolbox and the GENIE image analysis tool (Leica Microsystems). The staining intensity was scored as 0 (no staining), 1 (weak staining), 2 (moderate staining), or 3 (strong staining), and the extent (percentage) of expression was determined (Fig. 1). The densities of cells expressing CD3, CD8, CD45RO, PD-1, and PD-L1 were evaluated using the Aperio nuclear algorithm (Fig. 1) and by counting the cells that were positive for these markers in 5 random square areas (1 mm^2^ each) in each of the intratumoral compartments. The average total number of cells that were positive for each marker in the 5 square areas was expressed in density per mm^2^. Density = cell count/mm^2^.

## 3 Results

### 3.1 Demographic and clinicopathological data

TMA blocks were created using CRC specimens obtained from a total of 421 primary CRC patients (181 female, 240 male) for analyses including PD-1, PD-L1, CD-3, CD-8, and CD45RO expression on ICs. The baseline demographics of these patients included smoking and alcohol consumption histories (Table 1) and also tumor clinicopathological characteristics (Table 2). The mean age was 59.0 years (SD 13.8 years), and 57% (240 of 421) of patients were males. The percentages of patients at each pathological stage were 7.6% at stage I, 64.6% at stage II, and 27.8% at stage III (Table 2).

**Table 1:** Demographic factors related to various markers of immune cells among 421 patients with colorectal cancer.

		PD-1% cell average			PDL-1% cell average			CD-8% cell average			CD-3% cell average			CD45RO % cell average		
	Overall	Mean	SD	*P*	Mean	SD	*P*	Mean	SD	*P*	Mean	SD	*P*	Mean	SD	*P*
Age	59.0 ± 13.8	*r* = –0.099^∗^		.345	*r* = –0.003		.676	*r* = 0.045		.468	*r* = –0.042		.536	*r* = –0.014		.002
Sex				.857			.988			.109			.614			.928
Female	181 (43.0%)	0.42	1.05		1.44	2.55		5.23	3.95		1.51	2.12		0.07	0.17	
Male	240 (57.0%)	0.40	0.86		1.10	1.60		5.64	3.72		1.43	1.83		0.10	0.30	
Family Hx				<.001			.552			.172			.104			.727
None	103 (24.5%)	0.23	0.33		1.10	1.63		5.93	3.98		1.37	2.14		0.07	0.22	
FAP	1 (0.2%)	0.11			0.88			3.39			0.50			0.03		
HNPCC	82 (19.5%)	0.87	1.45		1.37	1.98		5.24	3.31		1.57	1.90		0.10	0.30	
Positive FH	235 (55.8%)	0.33	0.88		1.26	2.40		5.33	3.41		1.42	2.18		0.08	0.27	
Smoking history				.528			.892			.006			.737			.456
Never	261 (62.0%)	0.42	0.93		1.31	2.21		5.11	3.80		1.44	1.99		0.10	0.28	
Ex-smoker	61 (14.5%)	0.37	0.90		1.08	1.78		6.52	4.63		1.71	2.31		0.06	0.16	
Current smoker	99 (23.5%)	0.40	1.02		1.18	1.84		5.77	3.20		1.37	1.59		0.07	0.21	
Alcohol consumption				.034			.362			.203			.540			.818
Never	282 (67.0%)	0.46	1.01		1.18	1.96		5.27	3.75		1.51	2.05		0.08	0.22	
Ex-drinker	29 (6.9%)	0.43	1.25		1.03	1.25		6.56	5.21		1.46	2.35		0.12	0.34	
Current drinker	110 (26.1%)	0.28	0.63		1.47	2.47		5.68	3.57		1.35	1.58		0.09	0.29	

**Table 2:** Clinicopathologic factors related to various markers of immune cells among 421 patients with colorectal cancer.

		PD-1% cell average			PDL-1% cell average			CD-8% cell average			CD-3% cell average			CD45RO% cell average		
	Overall	Mean	SD	*P*	Mean	SD	*P*	Mean	SD	*P*	Mean	SD	*P*	Mean	SD	*P*
Tumor location				<.001			.121			<.001			<.001			.585
Right colon	144 (34.2%)	0.67	1.33		1.63	2.77		6.40	4.21		2.04	2.66		0.06	0.09	
Left colon	146 (34.7%)	0.21	0.35		1.15	1.79		5.00	3.56		1.16	1.31		0.09	0.25	
rectum	129 (30.6%)	0.35	0.84		0.94	1.25		5.01	3.49		1.17	1.46		0.12	0.35	
Unclassified	2 (0.5%)	0.11	0.13		0.14	0.06		2.25	2.66		0.33	0.42		0.03	0.03	
Tumor_size	45.7 ± 477.4	*r* = 0.046		.345	*r* = –0.020		.767	*r* = –0.035		.468	*r* = –0.030		.536	*r* = 0.022		.652
Histology type				.027			.760			.287			.773			.217
Adenocarcinoma	369 (87.6%)	0.35	0.79		1.27	2.06		5.57	3.93		1.44	1.95		0.09	0.26	
Signet ring cell	3 (0.7%)	0.42	0.56		0.48	0.39		5.01	3.47		1.36	1.93		0.12	0.16	
Mucinous	42 (10.0%)	0.65	1.32		1.17	2.35		4.47	2.78		1.66	2.12		0.08	0.18	
Unclassified	7 (1.7%)	1.95	3.09		0.57	0.55		6.31	3.63		1.45	1.48		0.05	0.04	
Differentiation grade				<.001			.653			.243			.295			.399
Well	55 (13.1%)	0.33	0.74		1.38	2.30		5.44	3.69		1.78	2.08		0.14	0.43	
Moderate	298 (70.8%)	0.32	0.80		1.14	1.94		5.22	3.33		1.27	1.49		0.07	0.21	
Poor	64 (15.2%)	0.81	1.45		1.66	2.45		6.76	5.56		2.12	3.26		0.10	0.22	
Unclassified	4 (1.0%)	1.63	1.60		0.63	0.58		3.61	3.29		0.83	0.94		0.11	0.10	
TNM_T				.514			.002			.366			.018			.189
1	36 (8.6%)	0.35	0.58		2.35	3.44		4.89	3.20		1.93	1.61		0.09	0.14	
2	22 (5.2%)	0.75	1.55		2.51	2.78		4.88	3.43		1.66	1.45		0.04	0.06	
3	258 (61.4%)	0.42	1.01		1.21	2.04		5.66	3.76		1.43	1.91		0.09	0.26	
4	104 (24.8%)	0.22	0.37		0.90	1.44		5.25	4.30		1.35	2.25		0.04	0.09	
TNM_N				.976			.787			.505			.524			.536
0	312 (74.5%)	0.43	1.00		1.28	2.04		5.69	4.06		1.44	2.01		0.08	0.24	
1	59 (14.1%)	0.39	0.93		1.27	2.10		4.90	3.10		1.49	2.05		0.08	0.20	
2	42 (10.0%)	0.28	0.52		1.08	2.38		4.69	2.69		1.57	1.48		0.13	0.37	
3	6 (1.4%)	0.43	0.76		0.90	1.43		6.37	3.65		1.86	1.90		0.04	0.05	
TNM staging				.238			.359			.570			.239			.658
Stage I	22 (7.6%)	0.51	0.67		1.49	2.62		5.99	3.14		1.6 4	2.83		0.09	0.257	
Stage II	272 (64.6%)	0.40	0.97		1.29	2.06		5.69	4.14		1.44	2.03		0.06	0.17	
Stage III	113 (27.8%)	0.37	0.84		1.20	2.43		4.89	2.89		1.43	1.34		0.11	0.31	

Over a median follow-up duration of 43.5 months, 135 (32.1%) patients had relapsed and 120 (28.5%) patients had died.

### 3.2 Varied density of subtypes of ICs related to demographic factors

The densities (density = cell count/mm^2^) of CD3-, CD8-, CR45RO-, PD-1-, and PD-L1-expressing ICs in relation to demographic parameters are presented in Table 1.

Patients with a significantly higher density of PD-1-expressing ICs were more strongly correlated with Lynch syndrome patients than were those without a family history for this disease or other related diseases (*P* *<* .001). Additionally, there was a stronger correlation with patients who did not consume alcohol or had ceased alcohol consumption compared to that in patients that currently consume alcohol (*P* = .034).

Patients with a higher density of CD8-expressing ICs were significantly associated with current smoking habits or a smoking history (*P* = .006) comparing to associations with a nonsmoking history. A significant decrease in the density of CR45RO-expressing ICs was associated with aging (*P* = .002 and *r* = –0.014).

### 3.3 Varied density of subtypes of IC related to clinic-pathological features

As shown in Table 2, in patients with a higher density of CD3 and CD8 expression in addition to PD-1-expressing ICs, this expression pattern was significantly associated with the right colon (*P* < .001). A higher density of CD3-expressing ICs was also significantly associated with early tumor T staging (*P* = .018). A higher density of PD-1-expressing ICs is significantly correlated with mucinous type/signet ring cell adenocarcinoma (*P* = .027) and poor differentiation (*P* < .001). A higher density of PD-L1-expressing ICs was significantly associated with early tumor T staging (*P* = .002). There were no other statistically significant differences among the densities of the other subtypes of ICs in relation to clinical parameters.

### 3.4 Varied densities of subtypes of ICs were related to disease-free survival (DFS) and overall survival (OS) among stage I to III CRC patient

The clinicopathological factors related to DFS and OS are provided in Table 3. We next assessed if various IC markers were prognostic indicators for DFS and OS, and to assess this, univariate and multivariate Cox proportional-hazards analyses was performed among stage I to III patients. Univariate analysis revealed that patients with a higher density of PD-L1 ICs exhibited a significantly lower risk in regard to DFS (hazard ratio [HR]: 0.693, 95% confidence interval [CI]: 0.56–0.86, *P* *<* .001) and OS (HR: 0.813, 95% CI: 0.69–0.95, *P* = .011) (Table 4). Multivariate analysis further confirmed that patients possessing a higher density of PD-L1-expressing ICs exhibited significantly lower risks for recurrence and death after adjusting for age, smoking, alcohol consumption, tumor location, tumor histology, tumor grade, and tumor, lymph node and metastases (TNM) pathologic stage (DFS: adjusted HR: 0.752, 95% CI: 0.61–0.92, *P* = .006; OS: adjusted HR: 0.872, 95% CI: 0.75–1.91, *P* = .064) (Table 4). While higher densities of CD8+ ICs was also associated with a significantly lower risk in regard to recurrence (DFS: HR: 0.944, 95% CI: 0.89–1.00, *P* = .039) (Table 4), this lower risk was not significant after adjusting confounding factors (*P* = .372). There was no significant association between higher densities of CD3+, CD45RO+, or PD-1+ cells and disease recurrence or overall survival. (Table 4)

**Table 3:** The clinicopathologic factors related to DFS and OS.

	Disease free survival (DFS)			Overall survival (OS)		
	HR	(95% CI)	*P*	HR	(95% CI)	*P*
Age	0.999	(0.99–1.01)	.902	1.034	(1.02–1.05)	<.001
Sex (M vs. F)	0.943	(0.66–1.35)	.748	1.355	(0.93–1.97)	.110
Family Hx						
NONE	1			1		
FAP	5.107	(0.69–37.80)	.110	3.902	(0.53–28.82)	.182
HNPCC	1.043	(0.58–1.87)	.886	0.809	(0.45–1.47)	.486
Positive FH	1.306	(0.68–2.16)	.526	1.004	(0.49–1.67)	.747
Smoking history						
Never	1			1		
Ex-smoker	0.955	(0.56–1.62)	.865	1.631	(1.02–2.62)	.043
Current smoker	0.995	(0.65–1.53)	.983	1.120	(0.72–1.73)	.609
Alcohol consumption						
Never	1			1		
Ex-drinker	0.682	(0.30–1.56)	.364	1.288	(0.64–2.58)	.474
Current drinker	1.025	(0.68–1.53)	.905	1.217	(0.81–1.82)	.337
Tumor_size	1.000	(1.00–1.00)	.012	1.000	(1.00–1.00)	<.001
Tumor location						
1–3	1			1		
4–7	1.046	(0.67–1.64)	.843	0.975	(0.63–1.51)	.909
8	1.437	(0.93–2.23)	.104	1.160	(0.74–1.81)	.512
0/9	11.213	(2.65–47.39)	.001	10.422	(2.49–43.63)	.001
Histology type						
1	1			1		
2	3.278	(0.81–13.30)	.097	5.448	(1.34–22.23)	.018
3	1.069	(0.60–1.90)	.819	0.733	(0.37–1.45)	.370
5/7/9	1.036	(0.26–4.20)	.961	1.410	(0.45–4.45)	.558
Differentiation grade						
Well	1			1		
Moderate	1.918	(0.97–3.81)	.063	1.394	(0.76–2.55)	.281
Poor	2.630	(1.22–5.66)	.013	1.555	(0.75–3.20)	.231
Unclassified	4.298	(0.93–19.91)	.062	2.754	(0.61–12.33)	.185
TNM_T						
1	1			1		
2	2.287	(0.42–12)	.340	0.684	(0.16–2.86)	.684
3	3.370	(0.83–14)	.090	1.390	(0.56–3.46)	0.745
4	7.367	(1.79–30)	.006	2.737	(1.08–6.91)	0.041
TNM_N						
0	1			1		
1	1.533	(0.91–2.58)	.107	1.457	(0.88–2.42)	.146
2	4.286	(2.75–6.67)	<.001	2.675	(1.66–4.32)	<.001
3	11.132	(4.75–26.10)	<.001	5.947	(2.40–14.76)	<.001
TNM staging						
I/II	1			1		
III	1.678	(1.07–2.62)	.023	1.369	(0.86–2.18)	.184

**Table 4:** Unadjusted and adjusted hazard ratios of DFS and OS for varied immune cell subsets.

		Disease free survival (DFS)			Overall survival (OS)		
		Estimate	(95% CI)	*P*	Estimate	(95% CI)	*P*
PD-1% cell average	HR	0.748	(0.54–1.04)	.080	0.729	(0.51–1.03)	.076
	aHR	0.832	(0.59–1.17)	.286	0.876	(0.64–1.19)	.400
PDL-1% cell average	HR	0.693	(0.56–0.86)	<.001	0.813	(0.69–0.95)	.011
	aHR	0.752	(0.61–0.92)	.006	0.872	(0.75–1.01)	.064
CD-8% cell average	HR	0.944	(0.89–1.00)	.039	0.996	(0.95–1.05)	.888
	aHR	0.973	(0.92–1.03)	.372	1.025	(0.97–1.08)	.365
CD-3% cell average	HR	0.892	(0.78–1.01)	.083	1.009	(0.91–1.11)	.862
	aHR	0.916	(0.80–1.04)	.191	1.037	(0.95–1.14)	.440
CD45RO% cell average	HR	1.082	(0.54–2.17)	.823	1.032	(0.51–2.10)	.931
	aHR	1.236	(0.61–2.49)	.555	1.452	(0.69–3.03)	.321

## 4 Discussion

In this study, we demonstrated that varied densities of ICs possessing different molecular markers significantly correlated with the demographic and clinicopathological factors and treatment outcomes in patients with nonmetastatic (stages I–III) CRCs.

Regarding oncologic outcomes, our results indicated that among various IC subsets, only those expressing a high density of PD-L1 served as an independent factor for improved prognosis of CRC. Similar to the previous study findings,^[6,7,11–13]^ we found that a high density of PD-LI ICs correlated with improved DFS, using multivariate analysis after adjustment for age, sex, TNM stage, tumor location, and differentiation grade. These findings were applied to this cohort (HR = 0.752; 95% CI: 0.61–0.92, *P* = .006) and were also associated with borderline overall survival (HR = 0.872; 95% CI: 0.75–1.01, *P* = .064).

Although PD-L1 expression in colon cancer has been extensively investigated, inconsistent results have been reported. The discrepancies in PD-L1 expressions in different studies may be related to heterogeneous patient subgroups recruited, such as the proportion of patients in different stage and different IHC methods, including the type of clone, scoring method, and cut-off values for positivity.

Some studies recruited heterogeneous subgroups of patients with rectal and colon cancer of stages I to IV.^[12–16]^ Some studies have been limited to early stage colon cancer^[11,17–19]^ and the present study, while others included only early colon cancers.^[17,19]^, and the present study. The contradiction resulting from the metastatic settings and the early stages might be due to temporal and spatial differences in the microenvironment and PD-L1 expression.^[20,21]^ Thus, in this study, we only collected early stage (stage I–III) colon cancer cases and excluded rectal and stage IV colon cancer cases to obtain homogeneous samples.

Some studies have demonstrated differences in PD-L1 expression between tumor cells and ICs. Droeser et al have demonstrated an association between PD-L1 expression and improved prognosis in CRC patients with a proficient mismatch repair protein,^[13]^ while Dunne et al have reported that poor CRC prognosis is associated with PD-L1 expression in cancer cells (CCs) and TILs.^[17]^ Furthermore, Koganemaru et al have shown a significant association of high PD-L1 expression in CCs with a poor prognosis and high PD-L1 expression in TILs with a good prognosis.^[11]^ Recently, Eriksen et al have observed that PD-L1 expression in tumor cells in stage II CRC does not provide any prognostic impact for either the entire population or microsatellite instability (MSI) subgroup patients.^[18]^ Based on these disparate findings, studies have suggested that PD-L1 expression is a negative prognostic factor, mainly due to its expression in tumor cells.^[15]^ Although PD-L1 expression in tumor cells is considered a mechanism of immune escape and an adverse prognostic factor in some malignancies, PD-L1 expression in ICs may exert a different impact.^[6]^ To make matters more complex, mismatch repair (MMR) status may differentially impact PD-L1 expression related to survival. In this study, similar to the findings of Noh et al,^[8]^ we found that a high density of PD-L1 expression correlated with hereditary non-polyposis colorectal cancer (HNPCC) patients and early tumor stage; however, the significance of PD-L1 expression in MMR-proficient and MMR-deficient patients is currently unclear. Droeser et al^[13]^ have demonstrated that a high PD-L1 expression in MMR-proficient CRC is correlated with improved overall survival, while Dunne et al^[17]^ have reported that PD-L1 expression is associated with a significantly better DFS in MMR-deficient patients. Lee et al^[15]^ have shown that improved recurrence-free survival is observed when the tumor exhibited low PD-L1 expression in combination with high levels of PD-1- and PD-L1-positive TILs. Furthermore, determining the evaluation of adjuvant chemotherapy in improving DFS or OS in relation to PD-L1 expression might prove challenging in the microsatellite stable and MSI subgroups of GI malignancies.^[17,19,22]^ The complex relationship between the prognosis for nonmetastatic CRC and PD-L1 expression was further clarified based on the results of our study.

Furthermore, using the IHC method for analyses, PD-L1 expression could vary depending on the related technical issues, including the type of clone, scoring method, and cut-off values for positivity. First, although PD-L1 is expressed on tumor cells and inflammatory ICs, PD-L1 expression in CRC is not frequently observed in tumor cells,^[23]^ and this might not be the case for all clones used for IHC staining. Accordingly, PD-L1 expression in our study with the SP142 clone mostly occurred in the ICs of the tumor-related stroma, with a particular sensitivity of expression in the ICs and not just any tumor cells.^[19,23]^ In addition, many previous studies used cut-off values of >5% or <5% to define the positive or negative expression of PD-L1; however, in this study, we used IC density of PD-L1 expression as a continuous variable to analyze the prognosis. This might reflect an accurate scenario. Notably, there is no established consensus on the scoring method and cut-off values defining positivity for comparisons. Therefore, contradictory results were observed in studies using different cut-off levels to determine the scoring method and PD-L1 positivity.

Several significant associations were identified between different IC subsets and clinicopathological factors. In agreement with the literature,^[7,16,19,24]^ we found that a high density of PD-1 ICs is significantly associated with right-sided tumor location, mucinous histology, poor tumor grade, and HNPCC patients. Additionally, a high density of PD-L1-expressing ICs was significantly associated with the early T stage (Table 2). In contrast, Yang et al^[29]^ reported that patients with high PD-L1 expression were associated with inferior tumor stage and vascular invasion negativity.^[25]^ Interestingly, we also observed that a significantly low density of PD-1 ICs was associated with individuals who consumed alcohol (*P* = .034) (Table 2). Furthermore, we found other significant associations between smoking and a high density of CD8-expressing ICs in current smokers than former smokers and nonsmokers (*P* = .022). It has been demonstrated that smoking is associated with the expression of CD8 on neutrophils and may fuel chronic neutrophil activation-mediated morbidities, such as atherothrombosis and cancer in lung cancer patients.^[26]^ Compared with the group of “never smokers,” a high density of CD8+ cells was associated with current or former smoking patients with CRC. In contrast, smoking status was not significantly associated with CD3+ and CD45RO+ cells. These findings suggest an interaction between smoking and immunity in colorectal carcinogenesis, especially for lower T-lymphocyte responses, which was in line with a previous report.^[27]^ These findings provide evidence that smoking consumption might affect treatment outcomes by modifying the subsets of ICs.

With regard to the correlation between PD-L1 and other subtypes of ICs, previous studies have shown that PD-L1 expression in ICs is related to a high density of CD8-expressing lymphocytes,^[13]^ as overexpression of PD-L1 could be induced by CD8+ ICs within the tumor microenvironment. Similar to previous studies,^[28]^ our study demonstrated that PD-L1 expression was associated with mucinous and poor cell differentiation and right-sided tumor location. Our data were in line with those of some recent studies. PD-L1 expression is associated with MSI and cytotoxic TILs, which are characteristics of Lynch syndrome and HNPCC.^[15,28]^ A recent study demonstrated different treatment outcomes with respect to OS and DFS with consensus molecular subgroup (CMS) subtypes. In the CMS2/CMS3 subgroup, PD-L1 expression significantly differentiated patients with good and poor prognosis for OS and time to relapse.^[19]^ In this study, we demonstrated that patients with a high density of CD8+ ICs exhibited a longer DFS than those with low density, using the univariate analysis (Table 4). Recently, Shibutani et al^[1]^ reported that the PD-1/CD8 ratio, rather than the absolute number of PD-1 + TILs, could serve as a useful prognostic marker for stage II/III CRC. However, the immunoscore obtained by measuring CD3+ and CD8+ cell densities in the tumor center and the invasive margin was also validated as an effective predictive and prognostic factor for stage II to III CRC.^[3]^

Some limitations of this study should be acknowledged while interpreting our results. Although computer-assisted image analysis might contribute to the reliable assessment of positive area percentage and IC density in CRC specimens,^[1]^ we focused on analyzing IC expression and not on tumor cells, as PD-L1 expression was primarily found in the ICs. We were also unable to evaluate the ratio of the tumor and IC densities. In this study, we evaluated a relatively small number of patients, and the study design was retrospective.

In conclusion, we found that a significantly varied density of IC subsets was related to distinct demographic and clinicopathological factors. More importantly, a high density of PD-L1-expressing ICs provided an independent favorable prognostic factor for DFS and OS among patients with stage I to III CRC.

## Author contributions

**Conceptualization:** Tse-ching Chen, Jy-Ming Chiang.

**Data curation:** Ya-Ting Kuo.

**Formal analysis:** Ya-Ting Kuo.

**Funding acquisition:** Jy-Ming Chiang.

**Investigation:** Ya-Ting Kuo, Chen-Chou Lai, Sum-Fu Chiang.

**Methodology:** Tse-ching Chen.

**Supervision:** Jy-Ming Chiang.

**Validation:** Chen-Chou Lai, Sum-Fu Chiang.

**Writing – original draft:** Ya-Ting Kuo.

**Writing – review & editing:** Chun-Kai Liao, Jy-Ming Chiang.
